# Chronic Granulomatous Disease-Like Presentation of a Child with Autosomal Recessive PKCδ Deficiency

**DOI:** 10.1007/s10875-022-01268-8

**Published:** 2022-05-18

**Authors:** Anna-Lena Neehus, Karen Tuano, Tom Le Voyer, Sarada L. Nandiwada, Kruthi Murthy, Anne Puel, Jean-Laurent Casanova, Javier Chinen, Jacinta Bustamante

**Affiliations:** 1 Laboratory of Human Genetics of Infectious Diseases, Necker Branch, Necker Hospital for Sick Children, 24 Boulevard du Montparnasse, INSERM U1163 Paris, France; 2 Paris Cité University, Imagine Institute, Paris, France; 3 Department of Pediatrics, Allergy and Immunology Division, The David Clinic, Baylor College of Medicine and Texas Children’s Hospital, The Woodlands, TX USA; 4 St. Giles Laboratory of Human Genetics of Infectious Diseases, Rockefeller Branch, The Rockefeller University, New York, NY USA; 5 Howard Hughes Medical Institute, New York, NY USA; 6 Department of Pediatrics, Necker Hospital for Sick Children, AP-HP, Paris, France; 7 Study Center for Primary Immunodeficiencies, Necker Hospital for Sick Children, AP-HP, Paris, France

**Keywords:** Chronic granulomatous disease, PKCδ, *Burkholderia*, *DHR assay*

## Abstract

**Background:**

Autosomal recessive (AR) PKCδ deficiency is a rare inborn error of immunity (IEI) characterized by autoimmunity and susceptibility to bacterial, fungal, and viral infections. PKCδ is involved in the intracellular production of reactive oxidative species (ROS).

**Material and Methods:**

We studied a 5-year old girl presenting with a history of *Burkholderia cepacia* infection. She had no history of autoimmunity, lymphocyte counts were normal, and no auto-antibodies were detected in her plasma. We performed a targeted panel analysis of 407 immunity-related genes and immunological investigations of the underlying genetic condition in this patient.

**Results:**

Consistent with a history suggestive of chronic granulomatous disease (CGD), oxidative burst impairment was observed in the patient’s circulating phagocytes in a dihydrorhodamine 123 (DHR) assay. However, targeted genetic panel analysis identified no candidate variants of known CGD-causing genes. Two heterozygous candidate variants were detected in *PRKCD:* c.285C > A (p.C95*) and c.376G > T (p.D126Y). The missense variant was also predicted to cause abnormal splicing, as it is located at the splice donor site of exon 5. TOPO-TA cloning confirmed that exon 5 was completely skipped, resulting in a truncated protein. No PKCδ protein was detected in the patient’s neutrophils and monocyte-derived macrophages. The monocyte-derived macrophages of the patient produced abnormally low levels of ROS, as shown in an Amplex Red assay.

**Conclusion:**

PKCδ deficiency should be considered in young patients with CGD-like clinical manifestations and abnormal DHR assay results, even in the absence of clinical and biological manifestations of autoimmunity.

**Supplementary Information:**

The online version contains supplementary material available at 10.1007/s10875-022-01268-8.

## Introduction

Protein kinase C delta (PKCδ) is a protein kinase that is ubiquitously expressed and has been implicated in various pathways involved in both innate and adaptive immunity [1–4]. Autosomal recessive (AR) PKCδ deficiency is a very rare inborn error of immunity (IEI), which has been reported in 17 patients from 10 unrelated kindreds worldwide [5–13]. Fifteen of these 17 patients presented clinical features of autoimmunity and had antinuclear antibodies (ANA) or anti-double-stranded DNA (anti-dsDNA) antibodies. Two patients presented no signs of autoimmunity and were negative for both ANA and anti-dsDNA antibodies [5]. Thirteen patients were diagnosed with early-onset systemic lupus erythematosus (SLE) and displayed photosensitivity, malar rash, alopecia, and hepatosplenomegaly. One patient developed B-cell lymphoproliferative disease and was diagnosed with autoimmune lymphoproliferative syndrome (ALPS) type III (OMIM #615,559) (8). A progressive decline in CD19^+^ B cells was frequently observed, with high proportions of CD21^low^ B cells and low proportions of memory B cells. Besides autoimmunity, most PKCδ-deficient patients presented with recurrent infections with various fungi, bacteria, or viruses [5–13]. Only one asymptomatic individual has been reported to date, and this lack of symptoms may be explained by his young age at diagnosis [5]. The susceptibility to fungal and bacterial infections of these patients has been shown to be partially due to the impaired production of reactive oxygen species (ROS) by the patients’ phagocytes and myeloid cells [5]. Defective ROS production by phagocytes is the main cellular phenotype observed in patients with chronic granulomatous disease (CGD), who present with susceptibility to bacterial and fungal infections [14–16]. PKCδ deficiency and CGD are both characterized by impaired ROS production by phagocytes, but they also overlap in clinical presentation in terms of susceptibility to infectious disease. We report here a new patient with AR PKCδ deficiency and no history of autoimmunity who was initially suspected to have CGD.

## Materials and Methods

### Targeted Panel Analysis

Targeted panel analysis was performed on whole blood with the Invitae primary immunodeficiency panel covering 407 primary immunodeficiency genes (https://www.invitae.com/en/physician/tests/08100/#info-panel-assay_information Invitae, San Francisco, CA, USA).

### DNA Extraction and Sanger Sequencing

The presence of the *PRKCD* variants was confirmed on genomic DNA extracted from whole blood or saliva samples with the DNeasy blood and tissue kit (#69,504, Qiagen, Hilden, Germany). Exons 4 and 5 of *PRKCD* were amplified with the specific primer pairs 5′–TGGGAAAACACTGGTGCAGA-3′ and 5′-TCACACCCAGGACATGTTGG-3′ and 5′-AGGCTGCCCTCACTGACCTTGTTC-3′ and 5′-TCACGTGCATACACTCTCTGAGCGC-3′, respectively, with the GoTaq DNA polymerase (#M3005, Promega, Madison, WI, USA). Sanger DNA sequencing was then performed with the Big Dye Terminator v3.1 sequencing kit (#4,337,455, Thermo Fisher Scientific, Waltham, MA, USA) and capillary electrophoresis on an ABI Prism 3700 (#A30469, Thermo Fisher Scientific).

### Targeted Mutagenesis and Transient Transfection

Site-directed mutagenesis was performed on the *PRKCD*-WT pCMV6 plasmid (#RC221652, OriGene) with the Pfu Ultra II Fusion HS DNA (#600,674, Agilent) polymerase as previously described [5]. The DNA was then digested with *Dpn*I (#R0176L, New England Biolabs). PCR products were purified with the NucleoSpin Gel and PCR Clean-up kit (#740,609.50, Machery-Nagel). For the deletion of exon 5, blunting and ligation were performed with the Quick Blunting (#E1201S, New England Biolabs) and Quick Ligation (#M2200S, New England Biolabs) kits. Plasmids were amplified in NEB-10 β competent *Escherichia coli* cells (#C3019H, New England Biolabs) and purified with the QIAprep Spin Miniprep kit (#27106X4, Qiagen). HEK293T cells were transiently transfected with 1 µg of plasmid DNA in the presence of X-tremeGene9 DNA transfection reagent (#6,365,809,001, Merck), in accordance with the manufacturer’s instructions.

### TOPO-TA Cloning

Total RNA was extracted with the Quick-RNA Microprep Kit (#R1051, Zymo Research) from monocyte-derived macrophages (MDMs) from the patient and a healthy, unrelated control. RNA was transcribed to generate cDNA with the SuperScript II Reverse Transcriptase (#18,064,014, Thermo Fisher Scientific) and full-length *PRKCD* was amplified with the GoTaq DNA Polymerase (Promega). PCR products were inserted into the pCR-4 TOPO vector (#K457502, Thermo Fisher Scientific) and amplified in NEB-10 β competent *E. coli* cells (New England Biolabs). We analyzed 60 colonies for both the patient and control, using M13 forward and reverse primers to amplify the inserted cDNA.

### Western Blotting

Whole-cell lysates of transfected human embryonic kidney (HEK) 293T cells, neutrophils, and stimulated MDMs were prepared in a modified radioimmunoprecipitation assay buffer (25 mM Tris–HCl pH 7.4, 150 mM NaCl, 1% NP-40 and 1 mM EDTA) supplemented with protease inhibitor cocktail (#5,892,970,001, Merck) and phosphatase inhibitor cocktail (#4,906,837,001, Merck), 0.1 mM dithiothreitol (DTT; #20,290, Thermo Fisher Scientific), and 1 mM PMSF (#10,837,091,001, Merck). Protein concentrations were determined by Bradford protein assay, and 30 µg of protein was subjected to SDS-PAGE in Criterion TGX 10% precast gels (#5,671,033, BIO-RAD). The resulting bands were transferred onto nitrocellulose membranes (#1,704,159, BIO-RAD) with the Trans-Blot Turbo Transfer System (#1,704,150, BIO-RAD). Membranes were blocked by incubation in 3% BSA in PBS-T and were then incubated with antibodies directed against PKCδ, p-PKCδ (T505; #9374, Cell Signaling Technology), p-PKCδ (S643; #9376, Cell Signaling Technology), p40^*phox*^ (#07–503, Merck), p-p40^*phox*^ (T154; #4311, Cell Signaling Technology), vinculin (#sc-73614, Santa Cruz), and GAPDH (#sc-47724, Santa Cruz). Proteins were visualized with the Clarity Western ECL substrate (#1,705,061, BIO-RAD) or SuperSignal West Femto (#34,096, Thermo Fisher Scientific), with ChemiDoc MP (Biorad). Images were analyzed with Imagine Lab 5.1 (Bio-Rad Laboratories).

### RT-qPCR

Gene expression was analyzed on 50 ng of cDNA with the TaqMan Universal PCR Master Mix (Thermo Fisher Scientific) and Taq-Man probes for *GUSB* (#4326320E, Thermo Fisher Scientific) and *PRKCD* (#Hs01090047_m1 and #Hs01090051_m1, Thermo Fisher Scientific). Normalization was performed for each sample with *GUSB* (ΔcT) and values are expressed as 2^−ΔcT^.

### Differentiation of MDMs

CD14^+^ cells were isolated from peripheral blood mononuclear cells (PBMCs) by positive selection with anti-CD14 MicroBeads (#1330–050-201, Miltenyi Biotec) according to the manufacturer’s instructions. The differentiation of CD14^+^ cells was promoted by culture in RPMI 1640 supplemented with 10% FCS and M-CSF (50 ng/mL; #216-MC, R&D Systems) for 7 days. The cells were then cultured in the presence of M-CSF and IL-4 (50 ng/mL; #204-IL, R&D Systems) for a further 7 days, to ensure complete differentiation. The medium was replaced every 2 to 3 days.

### ROS Production Assays

ROS production by neutrophils and monocytes was analyzed by incubating whole blood with dihydrorhodamine 123 (DHR) and catalase in the presence or absence of PMA. Red blood cells were lysed with BD FACS lysing solution (#349,202, BD) and stained with the following antibodies: CD16 APC-H7 (#561,306, BD), CD66b V450 (#561,649, BD), and CD45 V500-C (#655,873, BD). Analysis was performed on a BD FACSCanto II (BD). H_2_O_2_ production by MDMs with and without phorbol 12 myristate 13 acetate (PMA) stimulation (400 ng/mL) was assessed with the Amplex Red Kit (#A22188, Thermo Fisher Scientific) in Krebs–Ringer bicarbonate buffer (K40002, Merck). Briefly, 3 × 10^4^ cells were cultured in RPMI 1640 containing 10% FCS in 96-well flat-bottom plates. MDMs were primed with 1000 IU/mL human recombinant interferon gamma (IFN-γ) (Imukin, Boehringer Ingelheim) 16 h before the experiment. H_2_O_2_ release was quantified with a Victor X4 plate reader (Perkin Elmer).

### Whole-Blood Stimulation and ELISA for Cytokines

Whole blood samples were collected from healthy controls and the patients into heparin-containing collection tubes. Samples were diluted 1:2 in RPMI 1640 supplemented with 100 IU/mL penicillin and 100 µg/mL streptomycin (#15,140,122, Thermo Fisher Scientific). Samples were either incubated with medium alone, alone with BCG (*Mycobacterium bovis*-BCG, Pasteur substrain) at a MOI of 20, or with BCG plus human recombinant (rh) IL-12 (20 ng/ml; #219-IL-005/CF, R&D Systems), BCG plus IFN-γ (Imukin, Boehringer Ingelheim) for 48 h at 37 °C and 5% CO_2_. The supernatants were collected and assessed for their IL-12p40 (#DP400, R&D Systems) and IFN-γ (#DIF50, R&D Systems) content in accordance with the manufacturer’s protocol.

### Intracellular IFN-γ Production

IFN-γ production by human T cells was assessed by intracellular staining as previously described [17]. Briefly, PBMCs were seeded in RPMI 1640 supplemented with 10% FCS in the presence of brefeldin A (1:1000; #555,029; BD Bioscience) and were either left unstimulated or stimulated for 6 h in the presence of CD2/CD3/CD28 beads (1:40; #10,970, Stem Cell technologies). Cells were then harvested and stained for CD3-PE (1:50; # 12–0038-42, Thermo Fisher Scientific) in the presence of a viability marker (#L34957, Thermo Fisher Scientific), followed by fixation and permeabilization (#88–8824-00, Thermo Fisher Scientific). Cells were stained for intracellular IFN-γ-AF700 (1:100; #561,024, BD) and TNF-APC (1:50; # 130–117-531, Miltenyi) and analyzed on a Fortessa X-20 Cell Analyzer (BD).

## Results

### Case Report

**Fig. 1:**
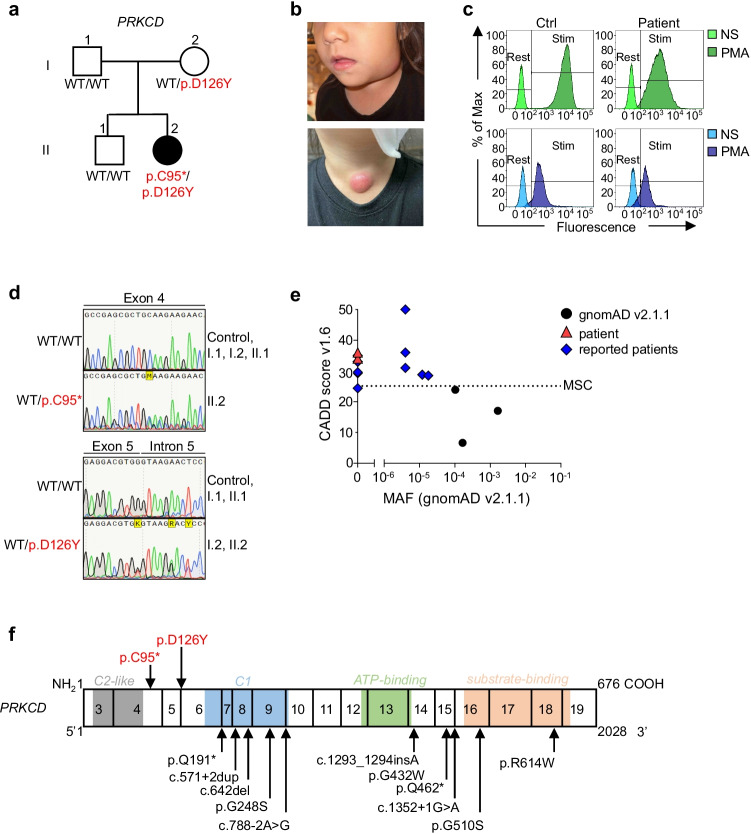
Genetic and clinical features of a patient with autosomal recessive PKCδ deficiency. **a** Pedigree of the family, showing familial segregation of the *PRKCD* alleles. Generations are indicated by Roman numerals (I–II), and each individual is indicated by an Arabic numeral (1–2). The patient is represented by closed black symbol. **b** Images of the patient presenting left submandibular (top) and left lower anterior cervical (bottom) lymphadenitis. **c** Flow cytometry images of intracellular ROS production in neutrophils (top) and monocytes (bottom) from a healthy control (Ctrl) and the patient before (NS) and after PMA stimulation. **d** Electropherogram of exons 4 and 5 showing the variants (p.C95* and D126Y) found in the patient and their comparison with a healthy control, the patient’s parents and brother. **e** Minor allele frequency (MAF) and combined annotation-dependent depletion (CADD) score of the heterozygous *PRKCD* variants (red triangles) found in the patient, variants reported in other PKCδ-deficient patients (blue lozenges) or found in the homozygous state in gnomAD v2.1.1 (black circles). The dotted line represented the mutation significant cutoff (MSC) with its 99% confidence interval. **f** Schematic representation of the PRKCD gene/protein. Coding exons are numbered from 3 to 19. The PKCδ protein is presented with the C2-like domain (gray), C1 domain (light blue), ATP-binding domain (green), and the substrate-binding domain (orange). Mutations reported in PKCδ-deficient patients are indicated in black and the two heterozygous mutations found in the patient are indicated in red

The patient (II.2) is a 5-year-old girl born to non-consanguineous parents originating from and living in the USA (Fig. 1a). Both parents and her older brother are healthy. She was vaccinated against diphtheria, tetanus and pertussis (DTaP), hepatitis A and B, *Haemophilus influenzae* Type b (Hib), and influenza and received a conjugated polysaccharide vaccine against S*taphylococcus pneumoniae* (PCV13) without adverse reaction. Vaccination with live vaccines against varicella and measles, mumps, and rubella (MMR) was also uneventful. She was not vaccinated with the live *Bacillus Calmette–Guérin* (BCG) vaccine. From the age of 2 years, the patient suffered from recurrent upper respiratory infections and multiple cervical lymphadenitis, presenting with a large submandibular lymph node. No fever, joint symptoms, malar rash, photosensitivity, or hepatosplenomegaly was reported. Fine-needle aspiration biopsy studies of the lymph node revealed an absence of granulomas, and cotrimoxazole-susceptible *Burkholderia cepacia* grew in culture. The lymphadenopathy resolved on oral cotrimoxazole therapy. One month later, the patient presented with another inflamed and enlarged lymph node, in the left lower anterior cervical chain (Fig. 1b). An excisional biopsy was performed and pathology studies reported an abscess without granuloma formation. Bacterial and fungal cultures were negative. Universal microbial PCR was positive for *Mycobacterium lentiflavum*. The patient was treated with cotrimoxazole, isoniazid, and rifampicin. Four months after the excision biopsy, her lymph nodes had returned to a minimal size and the patient was symptom-free. Whole-blood cell counts revealed normal counts of leukocytes, including polymorphonuclear neutrophils (PMNs) and monocytes, at several time points. Lymphocyte immunophenotyping by flow cytometry confirmed that the numbers of total T, B, and NK cells but decreased relative proportions of non-class-switched (CD27^+^IgD^+^) and class-switched (CD27^+^IgD^−^) memory B cells in the patient (Table 1; Fig. S1). Her proportion of CD21^low^ B cells was within normal range. At presentation, the patient tested negative for ANA, anti-dsDNA, anti-ribonucleoprotein and anti-Smith antibodies, and for autoantibodies against IFN-γ. An evaluation of the patient’s circulating phagocytes with the DHR assay revealed abnormally low levels of ROS production by PMNs and monocytes in response to PMA stimulation (Fig. 1c). Based on the patient’s clinical presentation and immunological findings, a diagnosis of CGD was suspected. However, a genetic analysis of CGD-associated genes — *CYBA*, *CYBB*, *NCF1*, *NCF2*, *NCF4* and *CYBC1* — was normal, and no copy-number variants were identified.

**Table 1:** Immunophenotyping in peripheral blood samples from the patient

		Patient	Normal range
T cells	CD3^+^ T cells (percentage of lymphocytes)	52.1	56.0–75.0
	CD4^+^ T cells (percentage of lymphocytes)	26.7	28.0–47.0
	CD8^+^ T cells (percentage of lymphocytes)	18.2	16.0–30.0
B cells	CD19^+^ (percentage of lymphocytes)	32.1	14–33.0
	CD21^low^CD38^dim^ (percentage of CD19^+^)	3.38	1.8–5.2
	CD27^- ^IgD^ +^ (percentage of CD19^+^)	98.3	76·3–84.9
	CD27^ +^ IgD ^+^ (percentage of CD19^+^)	0.25	4·1–9.0
	CD27^ +^ IgD^-^ (percentage of CD19^+^)	0.28	3·3–7.4
NK cells	NK cells (percentage of lymphocytes)	14.9	4.0–17.0

### Identification of Compound Heterozygous PRKCD Variants

The patient was investigated by target panel sequencing covering 407 immune system-related genes. Two heterozygous candidate variants of *PRKCD* were identified and predicted to be deleterious. One of the variants was a single-nucleotide substitution (c.285C > A) predicted to lead to a premature stop codon (p.C95*); the other was a single-nucleotide substitution (c.376C > T) leading to a missense variant (p.D126Y). The presence of the two variants and an AR pattern of inheritance were confirmed by Sanger sequencing (Fig. 1d). The p.D126Y variant was inherited from the mother, whereas the p.C95* variant was probably de novo. A combined annotation-dependent depletion (CADD) score of 34 was predicted for p.D126Y and of 36 for p.C95*. These values are well above the mutation significance cutoff (MSC), with a 99% confidence interval of 25.1 for *PRKCD*. The variants are private and have never been reported in public databases such as gnomADv2.1.1 and v3.1.2, ExAC, BRAVO/Topmed, or ATAV or in patients with AR PKCδ deficiency (Fig. 1e). Target panel sequencing also identified a private intronic variant, c.376 + 6A > G, in the same allele as p.D126Y. This variant had a low CADD score (15.28), was not predicted to abolish splicing, and is, therefore, probably not disease-causing. However, biallelic variants of *PRKCD* have been reported to underlie susceptibility to infections similar to those observed in patients with CGD [5]. Taken together, these results suggest that the patient is suffering from AR PRKδ deficiency.

### The Patient’s Variants Impair Splicing and Are Loss-of-Expression

**Fig. 2:**
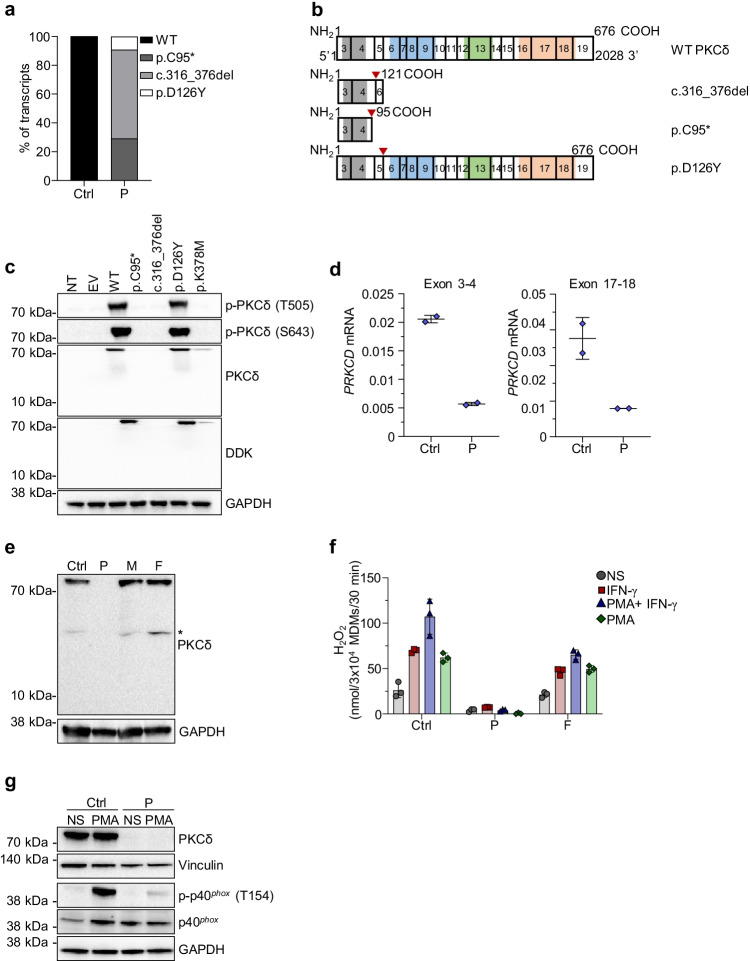
Functional analysis of the patient’s variants by overexpression and investigation of primary cells. **a** Proportions of the various *PRKCD* transcripts identified by TOPO-TA cloning on monocyte-derived macrophages (MDMs) from the patient (P) and a healthy control (Ctrl). **b** Schematic representation of the different splice variants found in P and their predicted consequences for the PKCδ protein. The positions of affected amino acids are indicated by red triangles. **c** Western blot of total cell extract from HEK293T cells not transfected (NT), or transfected with the pCMV6 empty vector (EV), wild-type (WT), or mutated *PRKCD* pCMV6. PKCδ phosphorylation was detected with antibodies against the phosphorylated p.T505 and p.S643 residues. **d** RT-qPCR for *PRKCD* on MDMs from a healthy control (Ctrl) and the patient (P), with probes spanning the junction between exons 3 and 4 (left) and exons 17 and 18 (right). *GUSB* was used for normalization (technical duplicates ± SD). **e** Western blot of total protein lysates isolated from polymorphonuclear neutrophils (PMNs) from a healthy control (Ctrl), the patient (P), her mother (M), and father (F). The asterisk indicates nonspecific bands. **f** Extracellular H_2_O_2_ release, measured in the Amplex Red assay after PMA stimulation with or without IFN-γ priming, for MDMs from a local control (Ctrl), the patient (P), and her WT/WT father (F) (technical triplicates ± SD). **g** Western blot on whole-cell lysates from MDMs of the patient (P) and a healthy control (Ctrl) not stimulated (NS) or stimulated with PMA for 15 min. The phosphorylation of p40^*phox*^ was assessed with an antibody against the phosphorylated p.T154 site

Both potentially deleterious variants are located in the N-terminal part of the PKCδ protein, between the C2-like and C1 domains (Fig. 1f). The p.D126Y variant is located at the end of exon 5 and is predicted, by GeneSplicer, NNSPLICE, and MaxEntScan, to disrupt the 5′ splice donor site. We studied the potential impact of the variants on mRNA splicing by performing TOPO-TA cloning and sequencing on cDNA from MDMs from the patient and a healthy control. Three different transcripts were detected for the patient, all of which were absent from the healthy control: 30% of the transcripts corresponded to the nonsense variant p.C95*, whereas 60% of transcripts displayed a skipping of exon 5 (c.316_376del) due to aberrant splicing and were predicted to encode a truncated protein (p.L106Ifs*17) (Fig. 2a and b). About 10% of transcripts corresponded to the p.D126Y variant, probably due to leaky splicing. Importantly, the variants were detected on different transcripts, confirming that the patient was compound heterozygous. We studied the impact of the different *PRKCD* transcripts by transiently transfecting HEK293T cells with plasmids encoding the wild-type (WT) or mutant *PRKCD* cDNAs, including a previously reported loss-of-function (LOF) mutant serving as negative control (p.K378M [9]). All constructs were C-terminally tagged with a Myc-DDK tag. Protein levels and basal activity were analyzed by western blotting whole-cell lysates. The WT, and the p.D126Y and p.K378M variants were produced at the expected molecular weight of 77 kDa, whereas the p.C95* and c.316_376del variants were undetectable with an antibody directed against the N-terminus of PKCδ (Fig. 2c). When an anti-DDK antibody was used, no protein was detected for the p.C95* and c.316_376del variants, excluding the possibility of a re-initiation of translation. Both variants are therefore loss-of-expression (LOE). Basal PKCδ activity was evaluated with antibodies against the autophosphorylated p.T505 and p.S643 residues. No autophosphorylation was observed at either of these residues, for the p.K378M negative control, whereas the p.D126Y variant presented normal autophosphorylation comparable to the WT protein. These results indicate that most of the transcripts identified in this patient are LOE when overexpressed.

### The Patient’s Phagocytes Display a Loss of PKCδ Expression and an Impaired Oxidative Burst

We then assessed the impact of the variants on mRNA and protein production in primary and MDMs from the patient. Quantitative PCR on MDMs with two different probes flanking the patient’s variants revealed that *PRKCD* mRNA levels in the patient were 75% decreased compared to those in a healthy control (Fig. 2d). PKCδ protein levels were analyzed by western blotting with whole-cell lysates from PMNs. No protein was detected in the cells of the patient, even at a lower molecular weight (Fig. 2e). Taken together, these results suggest that the patient’s mRNA is largely subjected to nonsense-mediated mRNA decay, resulting in an absence of detectable protein. We completed our study of the impact of the patient’s PKCδ variants on ROS production, by studying the NADPH oxidase activity of MDMs. Extracellular H_2_O_2_ release was evaluated in MDMs from the patient, a healthy control and the wild-type father, who served as a travel control. The patient displayed much lower levels of H_2_O_2_ production after PMA stimulation, in the presence or absence of IFN-γ priming, than the two controls (Fig. 2g). Furthermore, western blotting was performed on MDMs with and without PMA stimulation, to evaluate the PKCδ-dependent phosphorylation of p40^*phox*^ on the p.T154 residue. The absence of PKCδ protein was confirmed in the patient’s MDMs, together with abnormally low levels of p40^*phox*^ phosphorylation on p.T154 after PMA stimulation (Fig. 2h). Taken together, these results indicate that the patient’s circulating phagocytes and MDMs have impaired ROS production associated with a decrease in the phosphorylation of p40^*phox*^.

### Normal IFN-γ-Mediated Immunity in the Patient’s Cells

**Fig. 3:**
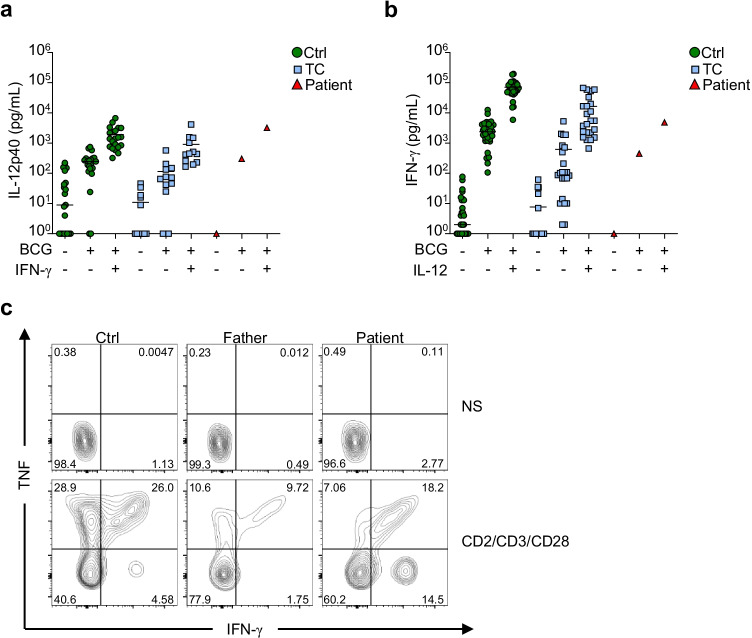
Conserved IFN-γ immunity in the PKCδ-deficient patient. **a** Secretion of IL-12p40 by whole blood from local controls (Ctrl; *n* = 21), travel controls (TC; *n* = 12), and the patient following the stimulation with BCG alone or in combination with IFN-γ. Cytokine levels were determined by ELISA. **b** Secretion of IFN-γ by whole blood from local controls (Ctrl; *n* = 30), travel controls (TC; *n* = 23), and the patient after stimulation with BCG alone or BCG and IL-12. Cytokine levels were determined by ELISA. **c** IFN-γ and TNF production by T cells from a local control (Ctrl), the patient and her WT/WT father with or without stimulation with CD2/CD3/CD28 beads

The patient presented with *M. lentiflavum* infection. Susceptibility to mycobacterial infections has been associated with an impaired or disrupted IFN-γ mediated immunity [17–19]. We studied the impact of AR PKCδ deficiency on the production of, or response to IFN-γ in vitro. The amounts of IL-12p40 secreted by whole blood of the patient in response to BCG or BCG plus IFN-γ were within the range of healthy controls (Fig. 3a). The secretion of IFN-γ was evaluated in whole blood of the patient following BCG or BCG plus IL-12 stimulation and was comparable to those observed in healthy controls (Fig. 3b). Additionally, we used conventional flow cytometry to analyze the production of IFN-γ and TNF in the patient’s primary T cells following stimulation with CD2/CD3/CD28 beads. The production of both cytokines in cells of the patient was comparable to those of the travel control (Fig. 3c). Collectively, these results indicate that AR PKCδ deficiency does not alter the global production or response to IFN-γ in vitro.

## Discussion

This newly identified patient with AR PKCδ deficiency presented with clinical and immunological findings common to the other reported patients — high susceptibility to infectious disease and impaired ROS production by various circulating phagocytes and MDMs — but no autoimmunity. She had *B. cepacia* infection, which is commonly seen in CGD patients [20, 21]. CGD can also be associated with the formation of granulomatous lesions [22]. While the patient we report here showed no granuloma formation, granulomatous inflammation in the lymph nodes has been reported in several PKCδ-deficient patients [5]. These observations highlight the overlap between the infectious and inflammatory phenotypes in patients with CGD and PKCδ deficiency. Interestingly, the patient also suffered from an infection caused by *M. lentiflavum*, a slow-growing environmental mycobacterium (EM) that is frequently isolated from water and soil samples [23]. Susceptibility to mycobacterial disease manifesting as BCG-itis after BCG vaccination has already been reported in four PKCδ-deficient patients [5], but this is the first such patient to present *M. lentiflavum* infection. Unlike *B. cepacia* infections, EM infections are rarely seen in patients with CGD and are more common in patients with Mendelian susceptibility to mycobacterial disease (MSMD) [19, 24, 25]. Patients with MSMD display a high susceptibility to EM due to genetic defects disrupting IFN-γ-mediated immunity [19]. Autoantibodies against IFN-γ are also known to underlie mycobacterial disease [26, 27], but no such antibodies were found in this patient. While some studies in mice and human cell lines suggest a role of PKCδ in host defense against *M. tuberculosis* and the upregulation of IFN-γ-dependent genes after IFN-γ stimulation, the production and response to IFN-γ was normal in cells of the patient [28, 29]. Susceptibility to mycobacterial disease has been previously associated with a defective ROS production in MDMs as shown in patients with X-linked recessive MSMD due to hypomorphic mutations in *CYBB* [30]*.* The defective ROS production by PKCδ-deficient MDMs could thus contribute to the susceptibility to mycobacterial infection observed in AR PKCδ deficiency. Strikingly, this patient presented no signs of autoimmunity at the time of diagnosis and had normal proportion of CD21^low^ B cells as well as no detectable auto-antibodies. Only two other PKCδ-deficient patients with no signs of autoimmunity have been described, one of whom had no history of infectious disease [5]. All three patients were below the age of 7 years, and the possibility of SLE development later in life cannot, therefore, be ruled out, but the mean age at onset of autoimmune manifestations in PKCδ-deficient patients is 3 years (± 2 years) [5, 8, 10, 11]. In conclusion, PKCδ deficiency can manifest clinically as a syndromic form resembling CGD. We suggest that *PRKCD* should be included in targeted panel analyses for the investigation of young patients presenting with a CGD-like infectious phenotype and abnormal DHR results.

## Supplementary Information

Below is the link to the electronic supplementary material.

Supplementary file1 (PDF 52 KB) Figure S1 B cell immunophenotyping. Representative flow cytometry images illustrating decreased numbers of class-switched and non-class-switched B cells in the patient when compared to her WT/WT father that served as travel control.

## Data Availability

All data are either included in the manuscript or are available upon request.
