# Supplementary figures and images for: Chronic Granulomatous Disease-Like Presentation of a Child with Autosomal Recessive PKCδ Deficiency
Source: J Clin Immunol. 2022 May 18;42(6):1244–53. doi: 10.1007/s10875-022-01268-8 (PMC9537221; doi:10.1007/s10875-022-01268-8)

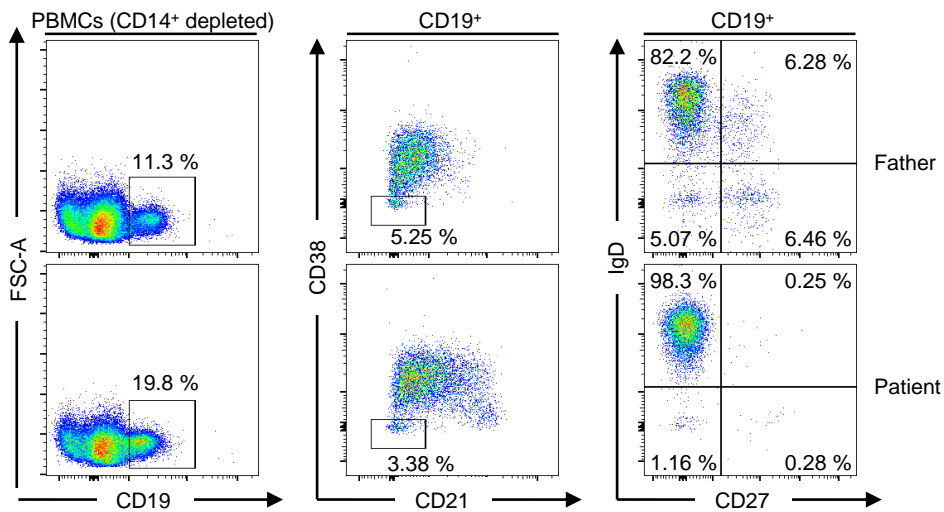

Figure S1

Supplement: Supplementary file 1 — Supplementary file1 (PDF 52 KB) Figure S1 B cell immunophenotyping. Representative flow cytometry images illustrating decreased numbers of class-switched and non-class-switched B cells in the patient when compared to her WT/WT father that served as travel control. [file 10875_2022_1268_MOESM1_ESM.pdf]
